# Supplementary material for: Description of a New miRNA Signature for the Surgical Management of Thyroid Nodules
Source: Cancers (Basel). 2024 Dec 18;16(24):4214. doi: 10.3390/cancers16244214 (PMC11674976; doi:10.3390/cancers16244214)
Supplement: Supplementary file 1 [file cancers-16-04214-s001.zip › cancers-3310534-supplementary.pdf]

SUPPLEMENTARY MATERIALS:

Supplementary methods:

S1: Quality controls:

The spike-ins are present in different concentrations in the mix and were used as quality controls of the RNA extraction, reverse-transcription and miRNA amplification steps, following the manufacturer recommendations. Total RNA obtained was evaluated for its quantity and quality using, respectively, a Nanodrop system (Isogen Life Science) and a bioanalyzer Experion (Bio-Rad). Amounts of 100 to 500 ng of total RNA per sample were reversely transcribed to cDNA using iScrip cDNA Synthesis Kit (Bio-Rad). The presence of follicular cells in the FNAB was controlled with a PAX8 gene expression assay using SsoAdvanced Universal SYBR Green Supermix following Bio-Rad recommendations. This gene encodes for one of the major transcription factors of thyroid follicular cells. Three different total RNA blood samples from healthy donors served as negative controls of PAX8 expression. As expected, no expression was detected in these samples.

S2: Molecular analyses:

An amount of 100 to 500 ng of total RNA per sample was reverse-transcribed to cDNA using iScript cDNA Synthesis Kit (Bio-Rad). Target mutational positions were amplified in PCR multiplex reaction using Platinum SuperFi DNA Polymerase (Thermo Fisher Scientific) following the recommendations. Specific cDNA primers that were designed to target the most prevalent point mutations and gene fusion detected in differentiated thyroid cancers were used [12,14,16]. These primers were designed using Primer 3 software (V.0.4.0) to generate oligonucleotides with similar characteristics, such as annealing temperature, GC content or length. Each primer pair was analyzed for its compatibility with other pairs for multiplexing PCR using the Multiple primer analyzer tool (Thermo Fisher Scientific). To decrease the cross-reaction between primer pairs, two PCR reactions were performed per sample: one targeting the point mutations and another targeting the fusions. Illumina adapter sequences were added to the 5′-ends of the primers, and they were synthetized by Eurogentec. Equal volumes of PCR products of both reactions were pooled per sample and quantified using Quant-iT PicoGreen kit following the recommendations (Thermo Fisher Scientific). Each sample was therefore 100-times diluted with nuclease-free water and used for tagging PCR. This step added sample-specific barcodes and instrument-specific adaptor sequences to the PCR product. An amount of 2.5 µl of each sample was mixed with 12.5 µl of Q5® High-Fidelity 2X Master Mix (New England BioLabs), 1, 25 µl of both NEXTERA XT index primers (Illumina) and 7.5 µl of nuclease-free water. After 15 cycles of 10 sec at 98°C, 30 sec at 65°C and 30 sec at 72°C and a final elongation step of 5 minutes at 72°C, tagging PCR products were purified and quantified using, respectively, the Agencourt AMPure XP beads (Beckman Coulter) and a Quant-iT PicoGreen kit following the manufacturer recommendations. An amount of 25 ng of each sample was pooled, and the quality of the final library obtained was controlled using a DNA 1000 kit on an Agilent 2100 Bioanalyzer system. The final library was sequenced by synthesis on a NovaSeq 6000 Illumina System (2X100 using a S2 flow cell) according to recommendations. Total RNA of control samples harboring each target mutation and non-RNA/cDNA controls were run with each batch of 30 samples. The mutation presented by the control samples were all confirmed by an orthogonal method (Sanger sequencing) as previously described.

S3: Analytical validation:

Minimal acceptable tumor cell content:

The lower limit of detection for the 7-Gene Mutation/Fusion Panel was estimated by diluting total RNA from 18 FNAB samples with known types of genetic alterations into a pool of 19 normal thyroid tissues previously characterized in our laboratory. Five serial dilutions were performed to achieve a theoretical variant allelic fraction (VAF) of +-3% for each point mutation.

Minimal acceptable tumor cell content in FNAB samples regarding correct miRNA classifier results was determined using total RNA from 3 cytologically indeterminate FNAB samples with known histopathological malignant diagnoses and reported as positive by our validated best algorithm

classifier. The corresponding nodules showed high tumor content (above 90%), and one of them presented a point mutation in the HRAS gene with a 50% VAF. A range of cancer cell concentrations from 100 to 0% were tested, diluting the total RNA from FNAB samples with the normal thyroid tissues pool.

Minimal acceptable thyroid cell content:

Similarly, the minimal acceptable thyroid cell content in FNAB samples regarding the validity of our miRNA classifier results was determined using the same 3 cytologically indeterminate FNAB samples. A range of thyroid cell concentrations from 100 to 0% were tested, diluting the total RNA from FNAB samples with a pool of 4 normal blood samples.

Assay precision:

To verify if the initial quantity could influence our results, 3 normal samples were diluted in 3 malignant samples by 75%, 50%, 25%, 12% and 6%. Our model was used to predict these values. None of the dilutions tested were wrongly predicted, suggesting that dilution of the original sample should not have a major impact (at tested ranges) on the predictive values of the classifier.

Figure S1: Boxplots for all raw Ct values for the 36 miRNAs, according to the status (malignant vs. benign).

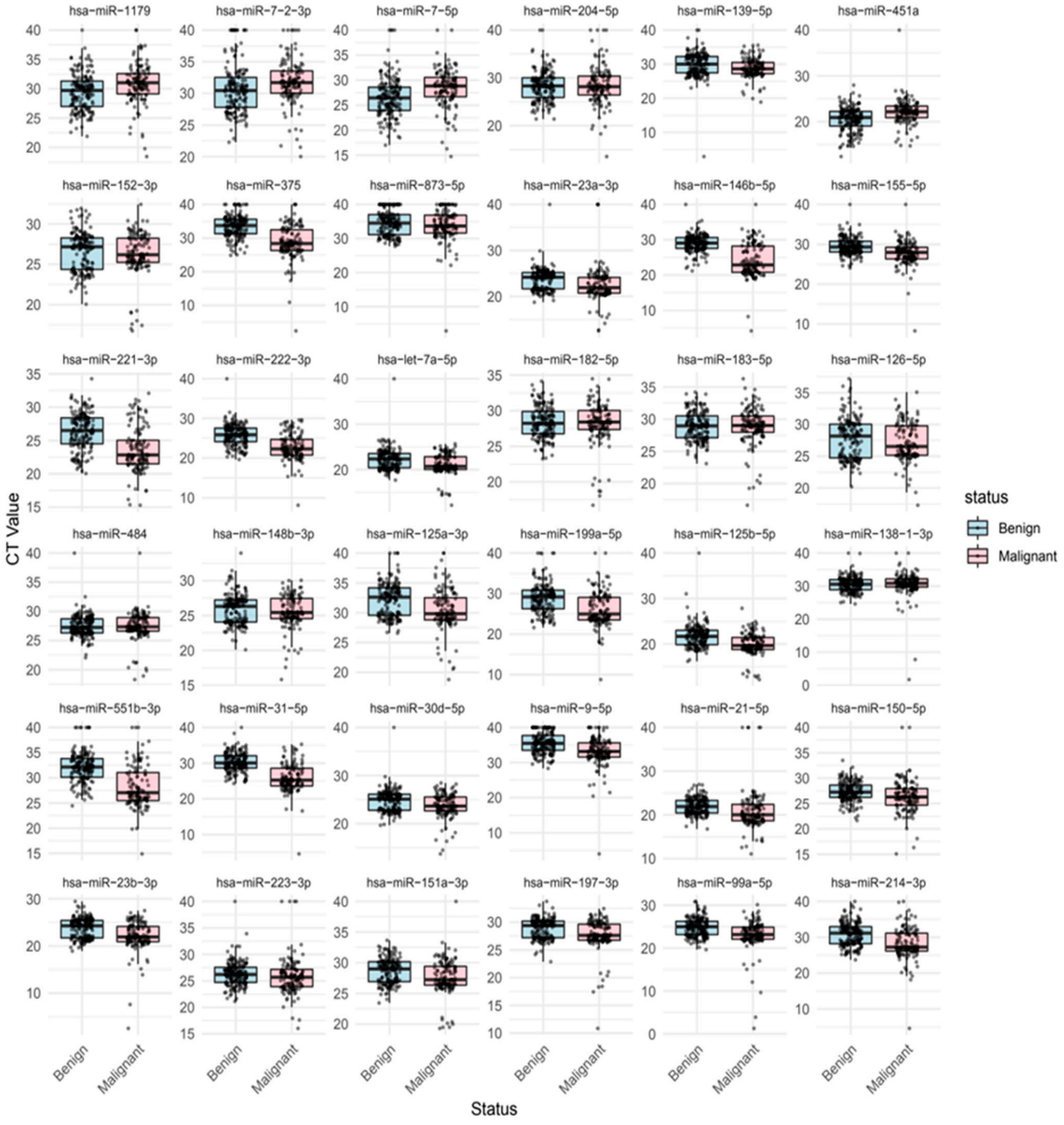

Table S1: Mutational status of the samples

Mutation	Malignant	Benign
BRAF	54	0
HRAS	1	6
NRAS	6	16
PAX8/PPARG	1	0
RET/PTC1	3	0
RET/PTC3	2	0
No mutation	58	147

Table S2: Complete list of molecular markers (delta "miRNA- minus-miRNA", point mutations and gene fusions) used by rf and classified by order of importance.

miRNA ratio	Overall Importance score [%]
hsa-miR-152-3p MINUS hsa-miR-125b-5p	100
hsa-miR-7-5p MINUS hsa-miR-125b-5p	81,97352749
hsa-miR-138-1-3p MINUS hsa-miR-551b-3p	78,7614946
hsa-miR-1179 MINUS hsa-miR-375	77,60581365
hsa-miR-1179 MINUS hsa-miR-146b-5p	76,50460258
hsa-miR-375 MINUS hsa-miR-148b-3p	75,36144824
hsa-miR-183-5p MINUS hsa-miR-31-5p	68,70066579
hsa-miR-152-3p MINUS hsa-miR-125a-3p	63,29240692
hsa-miR-7-5p MINUS hsa-miR-551b-3p	61,90150866
hsa-miR-7-2-3p MINUS hsa-miR-375	60,87288526
hsa-miR-7-5p MINUS hsa-miR-31-5p	59,13699714
hsa-miR-152-3p MINUS hsa-miR-99a-5p	57,92698588
hsa-miR-152-3p MINUS hsa-miR-31-5p	55,85005307
hsa-miR-7-2-3p MINUS hsa-miR-125b-5p	53,8945396
hsa-miR-7-5p MINUS hsa-miR-222-3p	53,2798023
hsa-miR-204-5p MINUS hsa-miR-214-3p	51,35311228
hsa-miR-138-1-3p MINUS hsa-miR-99a-5p	50,60608975
hsa-miR-148b-3p MINUS hsa-miR-551b-3p	49,69626868
hsa-miR-146b-5p MINUS hsa-miR-148b-3p	48,47967298
hsa-miR-7-2-3p MINUS hsa-miR-551b-3p	48,34545462

**hsa-miR-138-1-3p MINUS hsa-miR-31-5p**	**47,99261404**
**hsa-miR-148b-3p MINUS hsa-miR-125b-5p**	**47,67763381**
**hsa-miR-139-5p MINUS hsa-miR-31-5p**	**46,43700993**
**hsa-miR-222-3p MINUS hsa-miR-183-5p**	**46,11499471**
**hsa-miR-484 MINUS hsa-miR-31-5p**	**45,64005575**
**hsa-miR-1179 MINUS hsa-miR-125b-5p**	**44,41459799**
**hsa-miR-152-3p MINUS hsa-miR-199a-5p**	**43,99588002**
**hsa-miR-152-3p MINUS hsa-miR-551b-3p**	**43,21676704**
**hsa-miR-125b-5p MINUS hsa-miR-138-1-3p**	**42,70624042**
**hsa-miR-1179 MINUS hsa-miR-551b-3p**	**42,57106405**
**hsa-miR-7-5p MINUS hsa-miR-199a-5p**	**42,00328033**
**hsa-miR-1179 MINUS hsa-miR-222-3p**	**41,80120026**
**hsa-miR-148b-3p MINUS hsa-miR-31-5p**	**40,95458801**
**hsa-miR-375 MINUS hsa-miR-182-5p**	**39,31003157**
**hsa-miR-222-3p MINUS hsa-miR-30d-5p**	**39,12126815**
**hsa-miR-146b-5p MINUS hsa-miR-126-5p**	**39,07074015**
**hsa-miR-7-5p MINUS hsa-miR-375**	**37,18003594**
**hsa-miR-7-2-3p MINUS hsa-miR-214-3p**	**36,70390646**
**hsa-miR-7-5p MINUS hsa-miR-146b-5p**	**36,22130075**
**hsa-miR-183-5p MINUS hsa-miR-551b-3p**	**36,16497439**
**hsa-miR-7-2-3p MINUS hsa-miR-155-5p**	**35,27579244**
**hsa-miR-148b-3p MINUS hsa-miR-151a-3p**	**34,95635772**
**hsa-miR-7-2-3p MINUS hsa-miR-31-5p**	**33,77381931**
**hsa-miR-7-2-3p MINUS hsa-miR-199a-5p**	**33,72926317**
**hsa-miR-148b-3p MINUS hsa-miR-99a-5p**	**33,69032532**
**hsa-miR-1179 MINUS hsa-miR-99a-5p**	**33,34430678**
**hsa-miR-375 MINUS hsa-miR-138-1-3p**	**32,88581848**
**hsa-miR-126-5p MINUS hsa-miR-31-5p**	**32,34776586**
**hsa-miR-1179 MINUS hsa-miR-214-3p**	**32,0908669**
**hsa-miR-139-5p MINUS hsa-miR-375**	**31,87890332**
**hsa-miR-375 MINUS hsa-miR-183-5p**	**31,74312441**
**hsa-miR-222-3p MINUS hsa-miR-138-1-3p**	**31,39958883**
**statmut-rien**	**30,7730274**
**hsa-miR-7-5p MINUS hsa-miR-99a-5p**	**30,65679184**
**hsa-miR-138-1-3p MINUS hsa-miR-214-3p**	**30,45392166**
**hsa-miR-152-3p MINUS hsa-miR-23b-3p**	**29,81355294**
**hsa-miR-152-3p MINUS hsa-miR-23a-3p**	**29,43708853**
**hsa-miR-7-5p MINUS hsa-let-7a-5p**	**29,0909904**
**hsa-miR-148b-3p MINUS hsa-miR-214-3p**	**28,55142371**
**hsa-miR-7-5p MINUS hsa-miR-221-3p**	**28,24779728**
**hsa-miR-138-1-3p MINUS hsa-miR-21-5p**	**28,17426578**
**hsa-miR-152-3p MINUS hsa-miR-214-3p**	**27,41819153**
**hsa-miR-204-5p MINUS hsa-miR-31-5p**	**27,0077022**
**hsa-miR-126-5p MINUS hsa-miR-551b-3p**	**26,98874463**
**hsa-miR-873-5p MINUS hsa-miR-31-5p**	**26,83141819**
**hsa-miR-146b-5p MINUS hsa-miR-183-5p**	**26,79005145**

**hsa-miR-7-2-3p MINUS hsa-miR-99a-5p**	**26,51543175**
**hsa-miR-7-2-3p MINUS hsa-miR-139-5p**	**26,4613147**
**hsa-miR-7-2-3p MINUS hsa-miR-222-3p**	**25,98146762**
**hsa-miR-182-5p MINUS hsa-miR-31-5p**	**25,6446335**
**hsa-miR-148b-3p MINUS hsa-miR-23b-3p**	**25,44086635**
**hsa-miR-484 MINUS hsa-miR-551b-3p**	**24,70441698**
**hsa-miR-222-3p MINUS hsa-miR-148b-3p**	**24,54683559**
**hsa-miR-451a MINUS hsa-miR-146b-5p**	**24,46028199**
**hsa-miR-152-3p MINUS hsa-miR-146b-5p**	**23,85905817**
**hsa-miR-146b-5p MINUS hsa-miR-30d-5p**	**23,84205729**
**hsa-miR-7-2-3p MINUS hsa-miR-221-3p**	**23,80255145**
**hsa-miR-23a-3p MINUS hsa-miR-9-5p**	**23,69734462**
**hsa-miR-1179 MINUS hsa-miR-23a-3p**	**23,62110499**
**hsa-miR-7-2-3p MINUS hsa-miR-23a-3p**	**23,58777678**
**hsa-miR-7-5p MINUS hsa-miR-23a-3p**	**23,56554407**
**hsa-miR-7-5p MINUS hsa-miR-223-3p**	**23,50588748**
**hsa-miR-451a MINUS hsa-miR-125b-5p**	**23,29381414**
**hsa-miR-155-5p MINUS hsa-miR-221-3p**	**23,17723332**
**hsa-miR-1179 MINUS hsa-miR-199a-5p**	**23,12554711**
**hsa-miR-1179 MINUS hsa-miR-31-5p**	**23,09687523**
**hsa-miR-451a MINUS hsa-miR-222-3p**	**22,76460391**
**hsa-miR-1179 MINUS hsa-miR-151a-3p**	**22,54105064**
**hsa-miR-182-5p MINUS hsa-miR-551b-3p**	**22,48692285**
**hsa-miR-484 MINUS hsa-miR-214-3p**	**22,3258881**
**hsa-miR-451a MINUS hsa-miR-31-5p**	**22,19036346**
**hsa-miR-183-5p MINUS hsa-miR-125b-5p**	**22,14339054**
**hsa-miR-551b-3p MINUS hsa-miR-30d-5p**	**21,8141805**
**hsa-miR-155-5p MINUS hsa-miR-138-1-3p**	**21,59441488**
**hsa-miR-451a MINUS hsa-miR-214-3p**	**21,4853134**
**hsa-miR-451a MINUS hsa-miR-23a-3p**	**21,37007872**
**hsa-miR-7-5p MINUS hsa-miR-214-3p**	**21,26757591**
**hsa-miR-7-2-3p MINUS hsa-miR-150-5p**	**20,98804604**
**hsa-miR-23a-3p MINUS hsa-miR-31-5p**	**20,85882001**
**hsa-miR-375 MINUS hsa-miR-484**	**20,74485489**
**hsa-miR-148b-3p MINUS hsa-miR-30d-5p**	**20,45953495**
**hsa-miR-148b-3p MINUS hsa-miR-150-5p**	**20,40883269**
**hsa-miR-7-5p MINUS hsa-miR-9-5p**	**20,24378442**
**hsa-miR-375 MINUS hsa-miR-221-3p**	**20,23679628**
**hsa-miR-146b-5p MINUS hsa-miR-221-3p**	**20,13807465**
**hsa-miR-451a MINUS hsa-miR-9-5p**	**19,89288406**
**hsa-miR-222-3p MINUS hsa-miR-182-5p**	**19,86335477**
**hsa-miR-126-5p MINUS hsa-miR-21-5p**	**19,63210972**
**hsa-miR-152-3p MINUS hsa-miR-222-3p**	**19,43244436**
**hsa-miR-139-5p MINUS hsa-miR-551b-3p**	**19,42196513**
**hsa-miR-7-5p MINUS hsa-miR-155-5p**	**19,31392154**
**hsa-miR-204-5p MINUS hsa-miR-222-3p**	**19,29503292**

**hsa-miR-146b-5p MINUS hsa-miR-484**	**19,19294871**
**hsa-miR-183-5p MINUS hsa-miR-151a-3p**	**19,00878498**
**hsa-miR-1179 MINUS hsa-miR-221-3p**	**18,91029928**
**hsa-miR-199a-5p MINUS hsa-miR-30d-5p**	**18,89614472**
**hsa-miR-139-5p MINUS hsa-miR-9-5p**	**18,70996674**
**hsa-miR-125a-3p MINUS hsa-miR-138-1-3p**	**18,66263131**
**hsa-miR-125a-3p MINUS hsa-miR-31-5p**	**18,59350405**
**hsa-miR-1179 MINUS hsa-miR-150-5p**	**18,47562405**
**hsa-miR-146b-5p MINUS hsa-miR-125a-3p**	**18,43208159**
**hsa-miR-146b-5p MINUS hsa-miR-138-1-3p**	**18,36635456**
**hsa-miR-152-3p MINUS hsa-miR-155-5p**	**18,10166935**
**hsa-miR-221-3p MINUS hsa-miR-214-3p**	**18,03462842**
**hsa-miR-148b-3p MINUS hsa-miR-199a-5p**	**17,9531133**
**hsa-miR-125a-3p MINUS hsa-miR-551b-3p**	**17,93640481**
**hsa-miR-146b-5p MINUS hsa-miR-9-5p**	**17,92008896**
**hsa-miR-23a-3p MINUS hsa-miR-148b-3p**	**17,83817037**
**hsa-miR-7-2-3p MINUS hsa-miR-23b-3p**	**17,75509255**
**hsa-miR-484 MINUS hsa-miR-99a-5p**	**17,56386004**
**hsa-miR-375 MINUS hsa-miR-126-5p**	**17,43273368**
**hsa-miR-204-5p MINUS hsa-miR-551b-3p**	**17,26986173**
**hsa-miR-31-5p MINUS hsa-miR-23b-3p**	**17,11925389**
**hsa-miR-375 MINUS hsa-miR-150-5p**	**17,08479012**
**hsa-miR-551b-3p MINUS hsa-miR-223-3p**	**17,05100714**
**hsa-miR-126-5p MINUS hsa-miR-99a-5p**	**16,94810903**
**hsa-miR-1179 MINUS hsa-miR-125a-3p**	**16,8502447**
**hsa-miR-183-5p MINUS hsa-miR-21-5p**	**16,80245379**
**hsa-miR-183-5p MINUS hsa-miR-223-3p**	**16,6049696**
**hsa-miR-7-2-3p MINUS hsa-let-7a-5p**	**16,24936727**
**hsa-miR-183-5p MINUS hsa-miR-99a-5p**	**16,10727668**
**hsa-miR-873-5p MINUS hsa-miR-183-5p**	**16,05117943**
**hsa-miR-31-5p MINUS hsa-miR-30d-5p**	**16,01858085**
**hsa-miR-375 MINUS hsa-miR-873-5p**	**16,01141175**
**hsa-miR-182-5p MINUS hsa-miR-99a-5p**	**15,97133005**
**hsa-miR-148b-3p MINUS hsa-miR-9-5p**	**15,7916964**
**hsa-miR-9-5p MINUS hsa-miR-214-3p**	**15,74655587**
**hsa-miR-551b-3p MINUS hsa-miR-197-3p**	**15,71768872**
**hsa-miR-222-3p MINUS hsa-miR-31-5p**	**15,69794466**
**hsa-miR-146b-5p MINUS hsa-miR-182-5p**	**15,68822958**
**hsa-miR-152-3p MINUS hsa-miR-375**	**15,56760672**
**hsa-miR-484 MINUS hsa-miR-9-5p**	**15,54926319**
**hsa-miR-125b-5p MINUS hsa-miR-197-3p**	**15,47511436**
**hsa-miR-222-3p MINUS hsa-miR-484**	**15,26792458**
**hsa-miR-126-5p MINUS hsa-miR-125b-5p**	**15,25657685**
**hsa-miR-451a MINUS hsa-miR-551b-3p**	**15,24469886**
**hsa-miR-155-5p MINUS hsa-miR-126-5p**	**15,21640923**
**hsa-miR-7-2-3p MINUS hsa-miR-151a-3p**	**15,13911512**

**hsa-miR-199a-5p MINUS hsa-miR-23b-3p**	**14,81182348**
**hsa-miR-125b-5p MINUS hsa-miR-31-5p**	**14,71020844**
**hsa-miR-221-3p MINUS hsa-miR-222-3p**	**14,64909986**
**hsa-miR-148b-3p MINUS hsa-miR-197-3p**	**14,62830359**
**hsa-miR-375 MINUS hsa-miR-125a-3p**	**14,51819662**
**statmut-BRAF**	**14,45174839**
**hsa-miR-221-3p MINUS hsa-miR-125b-5p**	**14,38700351**
**hsa-miR-1179 MINUS hsa-let-7a-5p**	**14,20343458**
**hsa-miR-139-5p MINUS hsa-miR-199a-5p**	**14,19623871**
**hsa-miR-451a MINUS hsa-miR-151a-3p**	**14,18898111**
**hsa-miR-31-5p MINUS hsa-miR-150-5p**	**14,16597282**
**hsa-miR-148b-3p MINUS hsa-miR-21-5p**	**14,16543376**
**hsa-miR-199a-5p MINUS hsa-miR-551b-3p**	**14,12801**
**hsa-miR-21-5p MINUS hsa-miR-99a-5p**	**14,11549247**
**hsa-miR-1179 MINUS hsa-miR-204-5p**	**14,08009705**
**hsa-miR-873-5p MINUS hsa-miR-214-3p**	**13,99549712**
**hsa-miR-151a-3p MINUS hsa-miR-214-3p**	**13,93912832**
**hsa-miR-183-5p MINUS hsa-miR-30d-5p**	**13,91945773**
**hsa-miR-23a-3p MINUS hsa-miR-150-5p**	**13,85708587**
**hsa-miR-152-3p MINUS hsa-miR-221-3p**	**13,84085032**
**hsa-miR-551b-3p MINUS hsa-miR-9-5p**	**13,74043908**
**hsa-miR-375 MINUS hsa-miR-30d-5p**	**13,61809247**
**hsa-miR-125b-5p MINUS hsa-miR-9-5p**	**13,53574428**
**hsa-miR-146b-5p MINUS hsa-miR-125b-5p**	**13,48303033**
**hsa-miR-204-5p MINUS hsa-miR-375**	**13,43138608**
**hsa-miR-125b-5p MINUS hsa-miR-551b-3p**	**13,42104696**
**hsa-miR-7-2-3p MINUS hsa-miR-223-3p**	**13,42000956**
**hsa-miR-451a MINUS hsa-miR-21-5p**	**13,40725142**
**hsa-miR-155-5p MINUS hsa-miR-148b-3p**	**13,40374677**
**hsa-miR-182-5p MINUS hsa-miR-125b-5p**	**13,29287177**
**hsa-miR-125a-3p MINUS hsa-miR-199a-5p**	**13,21748381**
**hsa-miR-221-3p MINUS hsa-miR-183-5p**	**13,18572734**
**hsa-miR-139-5p MINUS hsa-miR-214-3p**	**13,01285772**
**hsa-miR-484 MINUS hsa-miR-125b-5p**	**12,8744307**
**hsa-miR-7-5p MINUS hsa-miR-21-5p**	**12,84018127**
**hsa-miR-126-5p MINUS hsa-miR-138-1-3p**	**12,79518025**
**hsa-miR-375 MINUS hsa-miR-23a-3p**	**12,79455269**
**hsa-miR-155-5p MINUS hsa-miR-222-3p**	**12,78145204**
**hsa-miR-155-5p MINUS hsa-miR-484**	**12,71118807**
**hsa-miR-221-3p MINUS hsa-miR-197-3p**	**12,63995318**
**hsa-miR-152-3p MINUS hsa-miR-183-5p**	**12,57768272**
**hsa-miR-30d-5p MINUS hsa-miR-99a-5p**	**12,56466378**
**hsa-miR-155-5p MINUS hsa-miR-150-5p**	**12,56445487**
**hsa-miR-183-5p MINUS hsa-miR-9-5p**	**12,51512422**
**hsa-miR-152-3p MINUS hsa-miR-9-5p**	**12,50454287**
**hsa-miR-199a-5p MINUS hsa-miR-214-3p**	**12,47128978**

hsa-miR-221-3p MINUS hsa-miR-31-5p	12,42440515
hsa-miR-221-3p MINUS hsa-miR-551b-3p	12,31659066
hsa-miR-451a MINUS hsa-miR-155-5p	12,29122446
hsa-miR-182-5p MINUS hsa-miR-183-5p	12,18930381
hsa-miR-375 MINUS hsa-miR-222-3p	12,18363492
hsa-miR-199a-5p MINUS hsa-miR-138-1-3p	12,15434208
hsa-miR-126-5p MINUS hsa-miR-214-3p	12,13609209
hsa-miR-451a MINUS hsa-miR-221-3p	12,13095515
hsa-miR-146b-5p MINUS hsa-miR-197-3p	12,11729646
hsa-miR-139-5p MINUS hsa-miR-223-3p	12,10892093
hsa-miR-7-2-3p MINUS hsa-miR-197-3p	12,05679507
hsa-miR-375 MINUS hsa-miR-551b-3p	12,05149096
hsa-miR-146b-5p MINUS hsa-miR-150-5p	12,05046113
hsa-miR-873-5p MINUS hsa-miR-148b-3p	12,03455839
hsa-miR-221-3p MINUS hsa-miR-21-5p	12,01851551
hsa-miR-152-3p MINUS hsa-miR-21-5p	12,01499497
hsa-miR-148b-3p MINUS hsa-miR-138-1-3p	11,95662524
hsa-miR-221-3p MINUS hsa-miR-150-5p	11,91532548
hsa-miR-182-5p MINUS hsa-miR-23b-3p	11,89325745
hsa-miR-1179 MINUS hsa-miR-23b-3p	11,84435432
hsa-miR-1179 MINUS hsa-miR-197-3p	11,83295804
hsa-miR-551b-3p MINUS hsa-miR-99a-5p	11,74109163
hsa-miR-221-3p MINUS hsa-miR-199a-5p	11,72813265
hsa-miR-126-5p MINUS hsa-miR-23b-3p	11,71855534
hsa-miR-7-2-3p MINUS hsa-miR-9-5p	11,71066958
hsa-miR-146b-5p MINUS hsa-miR-23b-3p	11,57742015
hsa-miR-451a MINUS hsa-miR-23b-3p	11,54833967
hsa-miR-126-5p MINUS hsa-miR-30d-5p	11,46846861
hsa-miR-221-3p MINUS hsa-miR-99a-5p	11,39435939
hsa-miR-138-1-3p MINUS hsa-miR-9-5p	11,34342093
hsa-let-7a-5p MINUS hsa-miR-214-3p	11,31660966
hsa-miR-1179 MINUS hsa-miR-183-5p	11,31515718
hsa-miR-204-5p MINUS hsa-miR-9-5p	11,25377892
hsa-miR-126-5p MINUS hsa-miR-9-5p	11,19761844
hsa-miR-31-5p MINUS hsa-miR-9-5p	11,17161943
hsa-miR-151a-3p MINUS hsa-miR-197-3p	11,11595548
hsa-miR-139-5p MINUS hsa-miR-125b-5p	11,11334235
hsa-miR-139-5p MINUS hsa-let-7a-5p	11,10681393
hsa-miR-1179 MINUS hsa-miR-9-5p	11,05674824
hsa-miR-551b-3p MINUS hsa-miR-150-5p	11,02501287
hsa-miR-451a MINUS hsa-miR-375	10,94301661
hsa-miR-125b-5p MINUS hsa-miR-30d-5p	10,89171857
hsa-miR-125a-3p MINUS hsa-miR-9-5p	10,86859564
hsa-miR-183-5p MINUS hsa-miR-126-5p	10,84255259
hsa-miR-7-5p MINUS hsa-miR-150-5p	10,83449316
hsa-miR-1179 MINUS hsa-miR-21-5p	10,81474293

hsa-miR-221-3p MINUS hsa-miR-9-5p	10,79996655
hsa-miR-1179 MINUS hsa-miR-139-5p	10,79169095
hsa-miR-155-5p MINUS hsa-miR-151a-3p	10,78984319
hsa-miR-23a-3p MINUS hsa-miR-146b-5p	10,72912252
hsa-miR-155-5p MINUS hsa-miR-197-3p	10,7135919
hsa-miR-138-1-3p MINUS hsa-miR-150-5p	10,67800841
hsa-miR-155-5p MINUS hsa-miR-31-5p	10,63114855
hsa-miR-152-3p MINUS hsa-miR-148b-3p	10,61076643
hsa-miR-1179 MINUS hsa-miR-138-1-3p	10,60655946
hsa-miR-375 MINUS hsa-let-7a-5p	10,55396166
hsa-miR-23a-3p MINUS hsa-miR-197-3p	10,53101242
hsa-miR-155-5p MINUS hsa-miR-99a-5p	10,51355695
hsa-miR-125b-5p MINUS hsa-miR-214-3p	10,50822216
hsa-miR-375 MINUS hsa-miR-9-5p	10,45739947
hsa-miR-221-3p MINUS hsa-miR-138-1-3p	10,40137134
hsa-miR-222-3p MINUS hsa-miR-150-5p	10,35123838
hsa-miR-126-5p MINUS hsa-miR-151a-3p	10,34770752
hsa-miR-125a-3p MINUS hsa-miR-125b-5p	10,32356616
hsa-miR-222-3p MINUS hsa-miR-223-3p	10,32095134
hsa-miR-551b-3p MINUS hsa-miR-214-3p	10,30989301
hsa-miR-152-3p MINUS hsa-miR-30d-5p	10,27300594
hsa-miR-30d-5p MINUS hsa-miR-214-3p	10,26357358
hsa-miR-484 MINUS hsa-miR-125a-3p	10,2316292
hsa-miR-9-5p MINUS hsa-miR-99a-5p	10,21432004
hsa-miR-9-5p MINUS hsa-miR-150-5p	10,20296888
hsa-miR-551b-3p MINUS hsa-miR-23b-3p	10,19431021
hsa-miR-125b-5p MINUS hsa-miR-23b-3p	10,17854438
hsa-miR-125b-5p MINUS hsa-miR-223-3p	10,16109787
hsa-miR-375 MINUS hsa-miR-214-3p	10,07121446
hsa-miR-183-5p MINUS hsa-miR-199a-5p	10,0691718
hsa-miR-150-5p MINUS hsa-miR-23b-3p	10,06562764
hsa-miR-221-3p MINUS hsa-miR-484	10,04677507
hsa-miR-125a-3p MINUS hsa-miR-99a-5p	10,03973565
hsa-miR-551b-3p MINUS hsa-miR-31-5p	9,993358435
hsa-miR-1179 MINUS hsa-miR-223-3p	9,981195187
hsa-miR-451a MINUS hsa-miR-99a-5p	9,973900282
hsa-miR-484 MINUS hsa-miR-199a-5p	9,95770953
hsa-miR-139-5p MINUS hsa-miR-23a-3p	9,92577282
hsa-miR-873-5p MINUS hsa-miR-138-1-3p	9,913514056
hsa-miR-30d-5p MINUS hsa-miR-150-5p	9,877255948
hsa-miR-126-5p MINUS hsa-miR-148b-3p	9,864635793
hsa-miR-139-5p MINUS hsa-miR-873-5p	9,824702632
hsa-miR-182-5p MINUS hsa-miR-199a-5p	9,823903776
hsa-miR-139-5p MINUS hsa-miR-125a-3p	9,747092278
hsa-miR-199a-5p MINUS hsa-miR-9-5p	9,727418057
hsa-miR-484 MINUS hsa-miR-23b-3p	9,711206122

hsa-miR-125a-3p MINUS hsa-miR-223-3p	9,705015208
hsa-miR-199a-5p MINUS hsa-miR-125b-5p	9,698156557
hsa-miR-23a-3p MINUS hsa-miR-551b-3p	9,685177092
hsa-miR-873-5p MINUS hsa-miR-221-3p	9,651531367
hsa-miR-222-3p MINUS hsa-miR-21-5p	9,617630607
hsa-miR-204-5p MINUS hsa-miR-197-3p	9,603510179
hsa-miR-182-5p MINUS hsa-miR-9-5p	9,600752283
hsa-miR-375 MINUS hsa-miR-31-5p	9,585582102
hsa-miR-31-5p MINUS hsa-miR-197-3p	9,540251937
hsa-miR-125a-3p MINUS hsa-miR-214-3p	9,490701942
hsa-miR-9-5p MINUS hsa-miR-23b-3p	9,462421393
hsa-miR-7-5p MINUS hsa-miR-125a-3p	9,461470916
hsa-let-7a-5p MINUS hsa-miR-197-3p	9,455969468
hsa-miR-146b-5p MINUS hsa-miR-155-5p	9,425369162
hsa-miR-204-5p MINUS hsa-miR-146b-5p	9,386758534
hsa-miR-31-5p MINUS hsa-miR-223-3p	9,359690592
hsa-miR-31-5p MINUS hsa-miR-21-5p	9,33489324
hsa-miR-7-5p MINUS hsa-miR-197-3p	9,3289955
hsa-miR-23b-3p MINUS hsa-miR-223-3p	9,305810311
hsa-miR-139-5p MINUS hsa-miR-222-3p	9,243542474
hsa-miR-375 MINUS hsa-miR-155-5p	9,151374637
hsa-miR-9-5p MINUS hsa-miR-223-3p	9,052067278
hsa-miR-7-5p MINUS hsa-miR-204-5p	8,994597123
hsa-miR-23a-3p MINUS hsa-miR-221-3p	8,968848454
hsa-miR-1179 MINUS hsa-miR-155-5p	8,883138315
hsa-miR-484 MINUS hsa-miR-21-5p	8,881182796
hsa-miR-23a-3p MINUS hsa-miR-138-1-3p	8,876909376
hsa-miR-1179 MINUS hsa-miR-7-2-3p	8,86768477
hsa-miR-451a MINUS hsa-miR-183-5p	8,806507246
hsa-miR-7-5p MINUS hsa-miR-23b-3p	8,793960029
hsa-miR-451a MINUS hsa-miR-125a-3p	8,785917397
hsa-miR-451a MINUS hsa-miR-223-3p	8,749306844
hsa-miR-375 MINUS hsa-miR-23b-3p	8,744583081
hsa-miR-30d-5p MINUS hsa-miR-21-5p	8,707110784
hsa-miR-484 MINUS hsa-miR-150-5p	8,691973277
hsa-let-7a-5p MINUS hsa-miR-148b-3p	8,626824219
hsa-miR-7-2-3p MINUS hsa-miR-125a-3p	8,613096069
hsa-miR-152-3p MINUS hsa-miR-150-5p	8,577732263
hsa-miR-222-3p MINUS hsa-miR-151a-3p	8,56815415
hsa-miR-139-5p MINUS hsa-miR-155-5p	8,529313314
hsa-miR-7-5p MINUS hsa-miR-138-1-3p	8,517857965
hsa-miR-138-1-3p MINUS hsa-miR-23b-3p	8,510631331
hsa-miR-21-5p MINUS hsa-miR-150-5p	8,497516782
hsa-miR-183-5p MINUS hsa-miR-214-3p	8,442549592
hsa-let-7a-5p MINUS hsa-miR-9-5p	8,43418464
hsa-miR-7-5p MINUS hsa-miR-139-5p	8,428115453

hsa-miR-7-2-3p MINUS hsa-miR-146b-5p	8,427247391
hsa-miR-139-5p MINUS hsa-miR-484	8,363912934
hsa-miR-30d-5p MINUS hsa-miR-151a-3p	8,258492319
hsa-miR-126-5p MINUS hsa-miR-199a-5p	8,253580226
hsa-miR-873-5p MINUS hsa-miR-125b-5p	8,244551
hsa-miR-23a-3p MINUS hsa-miR-183-5p	8,21766803
hsa-miR-155-5p MINUS hsa-miR-9-5p	8,143122446
hsa-miR-139-5p MINUS hsa-miR-451a	8,120628756
hsa-miR-551b-3p MINUS hsa-miR-21-5p	8,114292711
hsa-miR-375 MINUS hsa-miR-146b-5p	8,092963339
hsa-miR-139-5p MINUS hsa-miR-152-3p	8,035111443
hsa-miR-23a-3p MINUS hsa-miR-23b-3p	8,014896132
hsa-miR-204-5p MINUS hsa-miR-155-5p	8,009996556
hsa-miR-155-5p MINUS hsa-let-7a-5p	7,923716729
hsa-miR-139-5p MINUS hsa-miR-23b-3p	7,91316918
hsa-miR-221-3p MINUS hsa-miR-23b-3p	7,908170342
hsa-miR-155-5p MINUS hsa-miR-125b-5p	7,885674191
hsa-miR-451a MINUS hsa-let-7a-5p	7,865293789
hsa-miR-199a-5p MINUS hsa-miR-197-3p	7,865118959
hsa-miR-7-2-3p MINUS hsa-miR-204-5p	7,85068493
hsa-miR-31-5p MINUS hsa-miR-151a-3p	7,822121374
hsa-miR-125b-5p MINUS hsa-miR-21-5p	7,820299612
hsa-miR-139-5p MINUS hsa-miR-183-5p	7,687369161
hsa-miR-139-5p MINUS hsa-miR-221-3p	7,674129647
hsa-miR-155-5p MINUS hsa-miR-21-5p	7,608372813
hsa-miR-155-5p MINUS hsa-miR-199a-5p	7,587290541
hsa-miR-139-5p MINUS hsa-miR-30d-5p	7,564074086
hsa-miR-146b-5p MINUS hsa-let-7a-5p	7,557556408
hsa-miR-146b-5p MINUS hsa-miR-31-5p	7,557242536
hsa-miR-7-5p MINUS hsa-miR-183-5p	7,547964395
hsa-miR-152-3p MINUS hsa-miR-126-5p	7,545350649
hsa-let-7a-5p MINUS hsa-miR-23b-3p	7,528941512
hsa-miR-7-2-3p MINUS hsa-miR-451a	7,519028514
hsa-miR-873-5p MINUS hsa-miR-150-5p	7,517380857
hsa-miR-222-3p MINUS hsa-miR-99a-5p	7,516742894
hsa-miR-222-3p MINUS hsa-miR-9-5p	7,511559236
hsa-miR-23b-3p MINUS hsa-miR-197-3p	7,490738541
hsa-miR-1179 MINUS hsa-miR-451a	7,468709225
hsa-miR-150-5p MINUS hsa-miR-223-3p	7,430110097
hsa-miR-146b-5p MINUS hsa-miR-21-5p	7,418090492
hsa-miR-125a-3p MINUS hsa-miR-151a-3p	7,417037947
hsa-miR-23a-3p MINUS hsa-miR-484	7,409241882
hsa-miR-23b-3p MINUS hsa-miR-99a-5p	7,343956751
hsa-miR-222-3p MINUS hsa-miR-551b-3p	7,328941936
hsa-miR-873-5p MINUS hsa-miR-9-5p	7,323435978
hsa-miR-204-5p MINUS hsa-miR-138-1-3p	7,303968606

**hsa-miR-7-5p MINUS hsa-miR-126-5p**	**7,288922239**
**hsa-miR-7-5p MINUS hsa-miR-151a-3p**	**7,264524741**
**hsa-miR-451a MINUS hsa-miR-126-5p**	**7,254886773**
**hsa-miR-873-5p MINUS hsa-miR-197-3p**	**7,230018859**
**hsa-miR-199a-5p MINUS hsa-miR-99a-5p**	**7,213069635**
**hsa-miR-1179 MINUS hsa-miR-7-5p**	**7,183163354**
**hsa-miR-183-5p MINUS hsa-miR-150-5p**	**7,146766103**
**hsa-miR-152-3p MINUS hsa-let-7a-5p**	**7,139548238**
**hsa-let-7a-5p MINUS hsa-miR-150-5p**	**7,123817221**
**hsa-miR-204-5p MINUS hsa-miR-23a-3p**	**7,051559235**
**hsa-miR-204-5p MINUS hsa-miR-223-3p**	**6,950221947**
**hsa-miR-9-5p MINUS hsa-miR-197-3p**	**6,942517801**
**hsa-miR-126-5p MINUS hsa-miR-197-3p**	**6,941974843**
**hsa-miR-125a-3p MINUS hsa-miR-21-5p**	**6,934913704**
**hsa-miR-221-3p MINUS hsa-miR-148b-3p**	**6,844006108**
**hsa-miR-204-5p MINUS hsa-miR-125b-5p**	**6,814602378**
**hsa-miR-484 MINUS hsa-miR-151a-3p**	**6,813769025**
**hsa-miR-223-3p MINUS hsa-miR-151a-3p**	**6,794219418**
**hsa-miR-221-3p MINUS hsa-miR-30d-5p**	**6,782946355**
**hsa-miR-204-5p MINUS hsa-miR-23b-3p**	**6,777030887**
**hsa-miR-1179 MINUS hsa-miR-148b-3p**	**6,771669138**
**hsa-let-7a-5p MINUS hsa-miR-125b-5p**	**6,764370556**
**hsa-miR-146b-5p MINUS hsa-miR-99a-5p**	**6,732201594**
**hsa-miR-7-2-3p MINUS hsa-miR-152-3p**	**6,71597728**
**hsa-miR-873-5p MINUS hsa-miR-182-5p**	**6,715604735**
**hsa-miR-873-5p MINUS hsa-miR-146b-5p**	**6,715176366**
**hsa-miR-1179 MINUS hsa-miR-152-3p**	**6,711305789**
**hsa-let-7a-5p MINUS hsa-miR-151a-3p**	**6,664106416**
**hsa-miR-150-5p MINUS hsa-miR-214-3p**	**6,642354074**
**hsa-miR-139-5p MINUS hsa-miR-182-5p**	**6,635875149**
**hsa-miR-451a MINUS hsa-miR-138-1-3p**	**6,572040933**
**hsa-miR-23a-3p MINUS hsa-miR-214-3p**	**6,513141979**
**hsa-miR-204-5p MINUS hsa-miR-152-3p**	**6,492887147**
**hsa-miR-375 MINUS hsa-miR-197-3p**	**6,456900321**
**hsa-miR-155-5p MINUS hsa-miR-30d-5p**	**6,446232917**
**hsa-miR-199a-5p MINUS hsa-miR-150-5p**	**6,425789821**
**hsa-miR-1179 MINUS hsa-miR-484**	**6,382949891**
**hsa-miR-155-5p MINUS hsa-miR-183-5p**	**6,36183191**
**hsa-let-7a-5p MINUS hsa-miR-223-3p**	**6,346884349**
**hsa-miR-182-5p MINUS hsa-miR-214-3p**	**6,313444978**
**hsa-miR-21-5p MINUS hsa-miR-197-3p**	**6,30362469**
**hsa-miR-155-5p MINUS hsa-miR-223-3p**	**6,286492496**
**hsa-miR-223-3p MINUS hsa-miR-214-3p**	**6,28287173**
**hsa-miR-138-1-3p MINUS hsa-miR-151a-3p**	**6,245987656**
**hsa-miR-155-5p MINUS hsa-miR-551b-3p**	**6,183750069**
**hsa-miR-204-5p MINUS hsa-miR-139-5p**	**6,157463514**

**hsa-miR-183-5p MINUS hsa-miR-148b-3p**	**6,13889147**
**hsa-miR-23a-3p MINUS hsa-miR-125b-5p**	**6,124688311**
**hsa-miR-7-5p MINUS hsa-miR-30d-5p**	**6,117213644**
**hsa-miR-7-5p MINUS hsa-miR-148b-3p**	**6,113327683**
**hsa-miR-873-5p MINUS hsa-miR-126-5p**	**6,081160395**
**hsa-miR-204-5p MINUS hsa-miR-199a-5p**	**6,077940326**
**hsa-miR-183-5p MINUS hsa-miR-125a-3p**	**6,067922418**
**hsa-miR-139-5p MINUS hsa-miR-151a-3p**	**6,03393927**
**hsa-miR-197-3p MINUS hsa-miR-214-3p**	**6,020369006**
**hsa-miR-484 MINUS hsa-miR-138-1-3p**	**6,014117493**
**hsa-miR-204-5p MINUS hsa-miR-148b-3p**	**6,000743632**
**hsa-miR-30d-5p MINUS hsa-miR-223-3p**	**5,991757983**
**hsa-miR-126-5p MINUS hsa-miR-223-3p**	**5,982813835**
**hsa-miR-873-5p MINUS hsa-miR-551b-3p**	**5,9809547**
**hsa-miR-155-5p MINUS hsa-miR-23b-3p**	**5,959460835**
**hsa-miR-222-3p MINUS hsa-miR-125a-3p**	**5,946072361**
**hsa-miR-31-5p MINUS hsa-miR-99a-5p**	**5,940413697**
**hsa-miR-221-3p MINUS hsa-miR-182-5p**	**5,928658829**
**hsa-miR-139-5p MINUS hsa-miR-146b-5p**	**5,92200869**
**hsa-let-7a-5p MINUS hsa-miR-551b-3p**	**5,890665253**
**hsa-miR-139-5p MINUS hsa-miR-150-5p**	**5,884110759**
**hsa-miR-7-2-3p MINUS hsa-miR-148b-3p**	**5,87242164**
**hsa-miR-139-5p MINUS hsa-miR-21-5p**	**5,862089055**
**hsa-miR-451a MINUS hsa-miR-152-3p**	**5,78663707**
**hsa-miR-30d-5p MINUS hsa-miR-9-5p**	**5,784727634**
**hsa-miR-1179 MINUS hsa-miR-873-5p**	**5,773036047**
**hsa-miR-7-5p MINUS hsa-miR-152-3p**	**5,752698943**
**hsa-let-7a-5p MINUS hsa-miR-199a-5p**	**5,725512835**
**hsa-miR-7-2-3p MINUS hsa-miR-126-5p**	**5,713445337**
**hsa-miR-873-5p MINUS hsa-miR-99a-5p**	**5,692467458**
**hsa-miR-204-5p MINUS hsa-miR-150-5p**	**5,691570941**
**hsa-miR-451a MINUS hsa-miR-199a-5p**	**5,664694066**
**hsa-miR-451a MINUS hsa-miR-30d-5p**	**5,660686176**
**hsa-miR-7-2-3p MINUS hsa-miR-183-5p**	**5,653440797**
**hsa-miR-23a-3p MINUS hsa-miR-199a-5p**	**5,65007572**
**hsa-miR-23a-3p MINUS hsa-miR-99a-5p**	**5,647409146**
**hsa-miR-873-5p MINUS hsa-miR-222-3p**	**5,622284615**
**hsa-miR-125a-3p MINUS hsa-miR-150-5p**	**5,617259167**
**hsa-miR-873-5p MINUS hsa-miR-23b-3p**	**5,571881155**
**hsa-miR-21-5p MINUS hsa-miR-223-3p**	**5,5504455**
**hsa-miR-126-5p MINUS hsa-miR-150-5p**	**5,538958255**
**hsa-miR-199a-5p MINUS hsa-miR-31-5p**	**5,512835374**
**hsa-miR-139-5p MINUS hsa-miR-138-1-3p**	**5,49006091**
**hsa-miR-146b-5p MINUS hsa-miR-151a-3p**	**5,489189137**
**hsa-miR-23a-3p MINUS hsa-miR-30d-5p**	**5,468953837**
**hsa-miR-221-3p MINUS hsa-miR-126-5p**	**5,452888446**

**hsa-let-7a-5p MINUS hsa-miR-484**	**5,432513759**
**hsa-miR-7-2-3p MINUS hsa-miR-7-5p**	**5,414707647**
**hsa-miR-222-3p MINUS hsa-miR-199a-5p**	**5,40515965**
**hsa-miR-23a-3p MINUS hsa-miR-21-5p**	**5,40264239**
**hsa-miR-375 MINUS hsa-miR-223-3p**	**5,383301292**
**hsa-miR-31-5p MINUS hsa-miR-214-3p**	**5,346346176**
**hsa-miR-7-5p MINUS hsa-miR-873-5p**	**5,34513539**
**hsa-miR-150-5p MINUS hsa-miR-99a-5p**	**5,315650946**
**hsa-miR-204-5p MINUS hsa-miR-126-5p**	**5,311036751**
**hsa-miR-484 MINUS hsa-miR-148b-3p**	**5,310603042**
**hsa-miR-484 MINUS hsa-miR-223-3p**	**5,30685773**
**hsa-miR-138-1-3p MINUS hsa-miR-223-3p**	**5,283379455**
**hsa-miR-551b-3p MINUS hsa-miR-151a-3p**	**5,26737842**
**hsa-miR-183-5p MINUS hsa-miR-484**	**5,229293309**
**hsa-miR-199a-5p MINUS hsa-miR-151a-3p**	**5,22898666**
**hsa-miR-23a-3p MINUS hsa-miR-126-5p**	**5,21454457**
**hsa-miR-23a-3p MINUS hsa-miR-155-5p**	**5,211918446**
**hsa-miR-484 MINUS hsa-miR-30d-5p**	**5,171567509**
**hsa-miR-222-3p MINUS hsa-miR-126-5p**	**5,169428564**
**hsa-miR-151a-3p MINUS hsa-miR-99a-5p**	**5,159414455**
**hsa-miR-146b-5p MINUS hsa-miR-199a-5p**	**5,142636638**
**hsa-miR-126-5p MINUS hsa-miR-125a-3p**	**5,139324717**
**hsa-miR-23b-3p MINUS hsa-miR-151a-3p**	**5,129159664**
**hsa-miR-199a-5p MINUS hsa-miR-223-3p**	**5,128751119**
**hsa-miR-197-3p MINUS hsa-miR-99a-5p**	**5,108632655**
**hsa-miR-1179 MINUS hsa-miR-182-5p**	**5,105691012**
**hsa-miR-451a MINUS hsa-miR-873-5p**	**5,092401033**
**hsa-miR-451a MINUS hsa-miR-150-5p**	**5,072980628**
**hsa-miR-23a-3p MINUS hsa-miR-125a-3p**	**5,048844575**
**hsa-miR-155-5p MINUS hsa-miR-125a-3p**	**5,027556499**
**hsa-miR-7-2-3p MINUS hsa-miR-873-5p**	**5,01251929**
**hsa-miR-7-2-3p MINUS hsa-miR-484**	**4,990143624**
**hsa-let-7a-5p MINUS hsa-miR-138-1-3p**	**4,987803047**
**hsa-miR-7-5p MINUS hsa-miR-484**	**4,986201621**
**hsa-miR-152-3p MINUS hsa-miR-223-3p**	**4,972181388**
**hsa-miR-146b-5p MINUS hsa-miR-214-3p**	**4,947124925**
**hsa-miR-222-3p MINUS hsa-miR-214-3p**	**4,915326207**
**hsa-miR-182-5p MINUS hsa-miR-151a-3p**	**4,816663264**
**hsa-miR-221-3p MINUS hsa-miR-223-3p**	**4,813301994**
**hsa-miR-222-3p MINUS hsa-miR-125b-5p**	**4,78987745**
**hsa-miR-451a MINUS hsa-miR-182-5p**	**4,780153966**
**hsa-miR-223-3p MINUS hsa-miR-197-3p**	**4,750337674**
**hsa-miR-873-5p MINUS hsa-miR-223-3p**	**4,738314378**
**hsa-miR-182-5p MINUS hsa-miR-21-5p**	**4,727893679**
**hsa-miR-182-5p MINUS hsa-miR-197-3p**	**4,724888267**
**hsa-miR-222-3p MINUS hsa-miR-197-3p**	**4,711981024**

hsa-miR-23a-3p MINUS hsa-let-7a-5p	4,694832076
hsa-miR-7-5p MINUS hsa-miR-451a	4,689545434
hsa-miR-375 MINUS hsa-miR-151a-3p	4,650221512
hsa-miR-99a-5p MINUS hsa-miR-214-3p	4,632207531
hsa-miR-23a-3p MINUS hsa-miR-182-5p	4,62664533
hsa-miR-30d-5p MINUS hsa-miR-197-3p	4,604661469
hsa-miR-9-5p MINUS hsa-miR-151a-3p	4,597708553
hsa-miR-873-5p MINUS hsa-let-7a-5p	4,597563475
hsa-miR-23a-3p MINUS hsa-miR-223-3p	4,564324364
hsa-miR-182-5p MINUS hsa-miR-484	4,536259053
hsa-let-7a-5p MINUS hsa-miR-126-5p	4,491554087
hsa-miR-152-3p MINUS hsa-miR-197-3p	4,479136606
hsa-miR-150-5p MINUS hsa-miR-197-3p	4,468363721
hsa-miR-873-5p MINUS hsa-miR-151a-3p	4,453584745
hsa-miR-139-5p MINUS hsa-miR-197-3p	4,423652646
hsa-miR-204-5p MINUS hsa-miR-30d-5p	4,402136283
hsa-miR-139-5p MINUS hsa-miR-148b-3p	4,401621011
hsa-miR-1179 MINUS hsa-miR-30d-5p	4,370796711
hsa-miR-7-2-3p MINUS hsa-miR-138-1-3p	4,355772563
hsa-miR-223-3p MINUS hsa-miR-99a-5p	4,350477727
hsa-miR-150-5p MINUS hsa-miR-151a-3p	4,336740201
hsa-miR-375 MINUS hsa-miR-199a-5p	4,313440524
hsa-miR-204-5p MINUS hsa-miR-125a-3p	4,303806245
hsa-miR-146b-5p MINUS hsa-miR-551b-3p	4,303667493
hsa-miR-221-3p MINUS hsa-miR-151a-3p	4,300078455
hsa-miR-152-3p MINUS hsa-miR-138-1-3p	4,254533494
hsa-miR-21-5p MINUS hsa-miR-23b-3p	4,247985144
hsa-miR-182-5p MINUS hsa-miR-150-5p	4,216647166
hsa-miR-204-5p MINUS hsa-miR-484	4,215668876
hsa-miR-21-5p MINUS hsa-miR-151a-3p	4,17073919
hsa-miR-139-5p MINUS hsa-miR-99a-5p	4,156555078
hsa-miR-21-5p MINUS hsa-miR-214-3p	4,136908824
hsa-miR-182-5p MINUS hsa-miR-223-3p	4,121244108
hsa-miR-484 MINUS hsa-miR-197-3p	4,114475529
hsa-miR-873-5p MINUS hsa-miR-23a-3p	4,105952813
hsa-let-7a-5p MINUS hsa-miR-31-5p	4,088933843
hsa-miR-183-5p MINUS hsa-miR-138-1-3p	4,054213544
hsa-miR-138-1-3p MINUS hsa-miR-197-3p	4,053328046
hsa-miR-375 MINUS hsa-miR-21-5p	4,046533543
hsa-miR-873-5p MINUS hsa-miR-125a-3p	4,04009408
hsa-let-7a-5p MINUS hsa-miR-182-5p	4,028538605
hsa-miR-182-5p MINUS hsa-miR-125a-3p	3,999504198
hsa-miR-222-3p MINUS hsa-miR-23b-3p	3,996081053
hsa-miR-152-3p MINUS hsa-miR-484	3,95429177
hsa-miR-23a-3p MINUS hsa-miR-151a-3p	3,941705706
hsa-miR-148b-3p MINUS hsa-miR-125a-3p	3,919598152

hsa-miR-125b-5p MINUS hsa-miR-99a-5p	3,910344292
hsa-miR-152-3p MINUS hsa-miR-873-5p	3,890911501
hsa-miR-204-5p MINUS hsa-miR-21-5p	3,870950059
hsa-miR-204-5p MINUS hsa-miR-183-5p	3,866213264
hsa-miR-873-5p MINUS hsa-miR-21-5p	3,842971455
hsa-miR-125b-5p MINUS hsa-miR-150-5p	3,826105315
hsa-miR-7-5p MINUS hsa-miR-182-5p	3,725194639
hsa-miR-148b-3p MINUS hsa-miR-223-3p	3,719801904
hsa-miR-375 MINUS hsa-miR-125b-5p	3,699006961
hsa-miR-183-5p MINUS hsa-miR-23b-3p	3,686514124
hsa-miR-155-5p MINUS hsa-miR-182-5p	3,676277511
hsa-miR-182-5p MINUS hsa-miR-30d-5p	3,644934053
hsa-miR-125a-3p MINUS hsa-miR-30d-5p	3,616694298
hsa-miR-873-5p MINUS hsa-miR-30d-5p	3,584041009
hsa-miR-146b-5p MINUS hsa-miR-222-3p	3,410597594
hsa-miR-152-3p MINUS hsa-miR-151a-3p	3,409492948
hsa-miR-182-5p MINUS hsa-miR-148b-3p	3,382649428
hsa-miR-152-3p MINUS hsa-miR-182-5p	3,379273702
hsa-miR-451a MINUS hsa-miR-148b-3p	3,36194126
hsa-miR-204-5p MINUS hsa-miR-99a-5p	3,326716616
hsa-miR-7-2-3p MINUS hsa-miR-30d-5p	3,303583838
hsa-miR-204-5p MINUS hsa-miR-873-5p	3,243765009
hsa-let-7a-5p MINUS hsa-miR-183-5p	3,24342309
hsa-miR-873-5p MINUS hsa-miR-155-5p	3,239380124
hsa-let-7a-5p MINUS hsa-miR-21-5p	3,090881873
hsa-miR-221-3p MINUS hsa-let-7a-5p	3,072126522
hsa-let-7a-5p MINUS hsa-miR-30d-5p	3,058905175
hsa-miR-7-2-3p MINUS hsa-miR-182-5p	3,05381589
hsa-miR-451a MINUS hsa-miR-484	3,028971998
hsa-miR-23b-3p MINUS hsa-miR-214-3p	2,998011744
hsa-miR-125b-5p MINUS hsa-miR-151a-3p	2,995134499
hsa-miR-7-2-3p MINUS hsa-miR-21-5p	2,990152097
hsa-miR-9-5p MINUS hsa-miR-21-5p	2,766544656
hsa-miR-183-5p MINUS hsa-miR-197-3p	2,740565564
hsa-miR-222-3p MINUS hsa-let-7a-5p	2,738634313
hsa-miR-204-5p MINUS hsa-miR-221-3p	2,736113829
hsa-miR-375 MINUS hsa-miR-99a-5p	2,686366981
hsa-miR-146b-5p MINUS hsa-miR-223-3p	2,588021363
hsa-miR-126-5p MINUS hsa-miR-484	2,482146342
hsa-miR-204-5p MINUS hsa-miR-182-5p	2,375509164
hsa-miR-30d-5p MINUS hsa-miR-23b-3p	2,369972383
hsa-miR-451a MINUS hsa-miR-197-3p	2,230230712
hsa-miR-125a-3p MINUS hsa-miR-23b-3p	2,187485135
hsa-let-7a-5p MINUS hsa-miR-99a-5p	2,102810488
hsa-miR-873-5p MINUS hsa-miR-199a-5p	2,045131104
hsa-miR-155-5p MINUS hsa-miR-214-3p	2,040100642

**hsa-miR-199a-5p MINUS hsa-miR-21-5p**	**2,002848593**
**hsa-miR-1179 MINUS hsa-miR-126-5p**	**1,948908076**
**hsa-miR-221-3p MINUS hsa-miR-125a-3p**	**1,868361917**
**hsa-miR-138-1-3p MINUS hsa-miR-30d-5p**	**1,854003529**
**hsa-miR-23a-3p MINUS hsa-miR-222-3p**	**1,838326138**
**hsa-miR-182-5p MINUS hsa-miR-126-5p**	**1,829171525**
**hsa-miR-204-5p MINUS hsa-miR-151a-3p**	**1,74680453**
**hsa-miR-139-5p MINUS hsa-miR-126-5p**	**1,672789771**
**hsa-miR-204-5p MINUS hsa-miR-451a**	**1,666284839**
**hsa-miR-204-5p MINUS hsa-let-7a-5p**	**1,604924751**
**hsa-miR-182-5p MINUS hsa-miR-138-1-3p**	**1,378518311**
**hsa-miR-125a-3p MINUS hsa-miR-197-3p**	**1,181587124**
**hsa-miR-873-5p MINUS hsa-miR-484**	**1,174202204**
**hsa-let-7a-5p MINUS hsa-miR-125a-3p**	**0,669566037**
**statmut-NRAS**	**0,463182153**
**statmut-RET-PTC1**	**0**
**statmut-RET-PTC3**	**0**
**statmut-HRAS**	**0**
**statmut-PAX8-PPARG**	**0**
**statmut-KRAS**	**0**
